# Dynamic landscape and evolution of m^6^A methylation in human

**DOI:** 10.1093/nar/gkaa347

**Published:** 2020-05-14

**Authors:** Hui Zhang, Xinrui Shi, Tao Huang, Xueni Zhao, Wanying Chen, Nannan Gu, Rui Zhang

**Affiliations:** 1 Key Laboratory of Gene Engineering of the Ministry of Education, State Key Laboratory of Biocontrol, School of Life Sciences, Sun Yat-Sen University, Guangzhou 510275, PR China; 2 RNA Biomedical Institute, Sun Yat-Sen Memorial Hospital, Sun Yat-Sen University, Guangzhou 510120, PR China

## Abstract

m^6^A is a prevalent internal modification in mRNAs and has been linked to the diverse effects on mRNA fate. To explore the landscape and evolution of human m^6^A, we generated 27 m^6^A methylomes across major adult tissues. These data reveal dynamic m^6^A methylation across tissue types, uncover both broadly or tissue-specifically methylated sites, and identify an unexpected enrichment of m^6^A methylation at non-canonical cleavage sites. A comparison of fetal and adult m^6^A methylomes reveals that m^6^A preferentially occupies CDS regions in fetal tissues. Moreover, the m^6^A sub-motifs vary between fetal and adult tissues or across tissue types. From the evolutionary perspective, we uncover that the selection pressure on m^6^A sites varies and depends on their genic locations. Unexpectedly, we found that ∼40% of the 3′UTR m^6^A sites are under negative selection, which is higher than the evolutionary constraint on miRNA binding sites, and much higher than that on A-to-I RNA modification. Moreover, the recently gained m^6^A sites in human populations are clearly under positive selection and associated with traits or diseases. Our work provides a resource of human m^6^A profile for future studies of m^6^A functions, and suggests a role of m^6^A modification in human evolutionary adaptation and disease susceptibility.

## INTRODUCTION

Chemical modifications on RNA have been recently appreciated as an important regulatory feature (1). Recent technological breakthroughs, driven mainly by the sequencing-based approaches, have enabled the genome-wide profiling of such RNA modifications, particularly the RNA deamination and methylation (2–6). However, except for A-to-I (adenosine to inosine) RNA editing, which is the predominant type of RNA deamination in animals (7–12), less is known about the dynamics and evolution of most RNA modifications.

m^6^A is one of the most prevalent internal modifications in mRNAs (2,13–16). It is present among eukaryotic species that range from yeast, plants, flies to mammals. m^6^A RNA methylation is catalyzed by a multicomponent methyltransferase complex, including METTL3, METTL14 and WTAP (17,18). It has a consensus motif RRACH (in which R represents A or G, and H represents A, C or U). m^6^A methylation regulates the splicing, expression, decay and translation of mRNAs (19–21), and plays crucial roles in various cellular pathways and processes such as cell differentiation, development and metabolism (15). To date, m^6^A has been identified in several thousand human protein-coding genes. Although m^6^A profile of many cultured human cell lines and fetal human tissues have been reported (22,23), we still have limited information about the global landscape and dynamics of m^6^A in adult human tissues.

It has been hypothesized that gene regulation, ranging from transcriptional processing to post-transcriptional regulation, has a central role in phenotypic evolution (24–26). Therefore, a fundamental question in biology is to understand how natural selection has shaped the evolution of gene regulation (27,28), including RNA modifications. Some studies have shown that m^6^A peaks, which typically span one to several hundred nt, are conserved between human and mouse (2) and the m^6^A peak regions have much higher sequence conservation scores than those of randomly selected regions (13). While others suggest that only 37% of the m^6^A peaks are conserved between human and rhesus macaque (29), and the sequence of m^6^A RAC central motif is only slightly conserved than the control RAC sites (30). In addition, a recent study suggested that most m^6^A sites in CDS regions are evolutionarily unconserved (31). However, those studies were limited in scope and scale, thus, a systematic investigation of the selection pressure on individual m^6^A sites is needed.

## MATERIALS AND METHODS

### Sample procurement

Samples of nine human adult tissues were obtained from Chinese Brain Bank Center (Wuhan, China). These tissues were collected post-mortem from individuals with no known medical history. The consent of human tissue samples using autopsy was obtained from the patients’ families. Samples were lysed and homogenized in TRIzol Reagent (Invitrogen) using Precellys evolution tissue homogenizer (Bertin). Total RNA was extracted using chloroform and isopropanol following the manufacturer's protocol. The quality of the total RNAs was determined by agarose gel electrophoresis and three biological replicates of RNA samples that with thick 28S and 18S ribosomal RNA (rRNA) gel bands at an approximate mass ratio of 2:1 were selected. These tissues are from five donors (N1–N5), including frontal cortex (N1–N3), cerebellum (N1–N3), heart (N–N3), liver (N1–N3), lung (N1, N3, N5), kidney (N1, N2, N5), spleen (N1, N2, N5), muscle (N2–N4) and testis (N1-N3). N1, male, age 39; N2, male, age 44; N3, male, age 47; N4, male, age 57; N5, male, age 44.

### m^6^A-seq library preparation

m^6^A immunoprecipitation and library construction were performed as described previously with some modification (2). In brief, samples were lysed and homogenized in TRIzol Reagent (Invitrogen) using Precellys evolution tissue homogenizer (Bertin). Total RNA was extracted using chloroform and isopropanol following the manufacturer's protocol. Polyadenylated mRNA was enriched from total RNA using GenElute mRNA miniprep kit (Sigma-Aldrich). RNA samples were fragmented in 1X Next Magnesium RNA Fragmentation Buffer (NEB) at 94°C for 5min and fragmented RNA was then cleaned up using ethanol precipitation. 10ng fragmented RNA was used to construct input control library with VAHTS Stranded mRNA-seq library prep kit (Vazyme). 15–40 ug fragmented RNA was further incubated with 5ug rabbit anti-m^6^A polyclonal antibody (Synaptic Systems, catalog number 202 003) in IPP buffer (150 mM NaCl, 0.1% Igepal CA-630, 10 mM Tris–HCl, Ph7.4) overnight at 4°C. The m^6^A-Ab mixture was then immunoprecipitated by incubation with protein-G magnetic beads (Thermo Fisher, pre-blocked with 0.5 mg ml^−1^ BSA at 4°C for 2h) at 4°C for another 2h. After washing with IPP buffer, bound RNA was competitively eluted from the beads with 0.5 mg ml^−1^*N*^6^-methyladenosine (Sigma-Aldrich), followed by ethanol precipitation. RNA was resuspended in 8 μl water and used for library construction. Libraries were sequenced on HiSeq X (Illumina) to produce paired-end 150 bp reads.

### MeRIP-seq of HEK293T cells

MeRIP-seq was performed as described previously (23). In brief, total RNA of HEK293T cells was extracted and fragmented in Next Magnesium RNA Fragmentation Buffer (NEB) at 94°C for 5 min. 10 ng fragmented RNA was used to construct the input library. 300 ug of fragmented RNA was incubated with 5 ug rabbit anti-m^6^A polyclonal antibody (Abcam, catalog number ab151230) overnight at 4°C. After stringent washing, bound RNA was eluted by competition with *N*^6^-methyladenosine (Sigma-Aldrich), followed by rRNA removal with QIAseq FastSelect RNA Removal Kits (Qiagen). Both the input and IP libraries were constructed using NEBNext Ultra II Directional RNA Library Prep Kit for Illumina (NEB). Libraries were sequenced on HiSeq X (Illumina) to produce paired-end 150 bp reads.

### METTL3 knockout cell generation

METTL3 knockout cell was generated via CRISPR/Cas9-induced mutagenesis. In brief, a sgRNA sequence (GCAGAAGCGGCGTGCAGAAC) was designed using CRISPR-ERA (http://CRISPR-ERA.stanford.edu). The sgRNA template oligonucleotide was synthesized and cloned into lentiCRISPR v2 plasmid (Addgene#52961). The plasmid was transfected into HEK293T cells. Transfected cells were selected using puromycin (1 μg /ml). Mutant clones were selected by Sanger sequencing. The loss of METTL3 protein expression was verified with the METTL3 antibody (Proteintech, catalog number 15073-1-AP) by western blot. The m^6^A levels of HEK293T wild type and METTL3 knockout cells were measured using EpiQuik m^6^A RNA Methylation Quantification Kit (Epigentek). In brief, polyadenylated RNA was separated from total RNA using Oligo dT Magnetic Beads (Vazyme). 300ng polyadenylated mRNA was used for m^6^A quantification following the manufacturer's protocol.

### m^6^A-seq data analysis

m^6^A peak identification was performed as previously described (32). In brief, we trimmed the adaptor and low quality reads using Cutadapt (33) (-e 0.3 –minimum-length 25; –trim-n -q 20,20) and fastx toolkit (fastx_trimmer -f 6; -t 5 -m 20). rRNA reads were then removed using SortMeRNA (34). Next, clean reads were mapped to the human genome (hg19) using TopHat2 (version 2.1.0) (35). Enriched peaks were identified by scanning each gene using 100-nt sliding windows, and calculating an enrichment score for each sliding window (winscore).

$$\begin{equation*}{\rm{winscore\;}} = {\rm{\;log}}2\left( {\frac{{{\rm{MeanWinIP}}/{\rm{MedianGeneIP}}}}{{{\rm{MeanWinControl}}/{\rm{MedianGeneControl}}}}} \right)\end{equation*}$$

MeanWinIP and MeanWinControl are the mean coverage for each window for immunoprecipitation and input control, respectively. MedianGeneIP and MedianGeneControl are gene median coverages for immunoprecipitation and input control, respectively. Windows with RPKM ≥ 10 in the IP sample, enrichment score ≥2 in genes with RPKM in the input sample ≥1 were defined as enriched windows. Last, only the peaks with winscore ≥2 in at least two samples of a tissue type were considered as real m^6^A peaks.

To generate the metagene profile of m^6^A site distribution across transcripts, we first determined the number of bins that need to be divided for a given gene based on relative lengths between 5′UTR, CDS and 3′UTR of the human transcriptome (GENCODE v26). The relative lengths between 5′UTR, CDS and 3′UTR are 10:50:40, thus for each gene, 10, 50 and 40 bins of equal length were made for 5′UTR, CDS and 3′UTR, respectively. Next, for each m^6^A site, we assigned it to the longest isoform of the corresponding gene and determined which bin it is located in. Last, the number of sites for each bin was summarized and the curve was fitted with polyfit.

The mapping statistics of all datasets were summarized in Supplementary Table S1.

### m^6^A peak call via exomePeak and MeTPeak

IP and input reads were mapped as described above. For each tissue type, the consistent peaks in all replicates were called using exomePeak (36) or MeTPeak (37) with default parameters.

### Tissue specificity analysis

For each site, we first calculated its average winscore in each tissue type. Next, we calculated its tissue specificity index tau (38) using the average winscore of each site:

$$\begin{equation*}{\rm{tau\;}} = \frac{{\mathop \sum \nolimits_{i = 1}^n 1 - \widehat {{x_i}}}}{{n - 1}}\;;\;\;\widehat {{x_i}} = \frac{{{x_i}}}{{\mathop {\max }\limits_{1 \le i \le n} {x_i}}}\;\end{equation*}$$

  ${x_i}$ is  the  average winscore  of  a site  in  tissue  i; n is the number of tissues.

Shared m^6^A sites were defined as sites with tau <0.15. Tissue-specific m^6^A sites were defined as sites with tau >0.6.

### m6A-RIP qPCR

RIP was performed as described above. Both the IP and input RNAs were reverse transcribed and the m^6^A marked mRNAs and NC (GAPDH) mRNA were detected by qPCR. The enrichment fold of IP versus input of each gene was calculated and normalized to NC. Primers were listed as follows:

ATP5C1-F GGGAGCTTCGGCGCAT

ATP5C1-R CGCGCGAGAGAACATGGTAG

DCTN1-F GCACGGTTCCTGACAAGTCTA

DCTN1-R GACACAGAATCCTGCTTGCC

PSMB4-F ATGGAAGCGTTTTTGGGGTC

PSMB4-R GAGTGGACGGAATGCGGTAA

SDHAF2-F GCCTTGCTTCCGGCTTCTTA

SDHAF2-R TGTCCATCACTTGAGGCAGG

GAPDH-F TGCCAAATATGATGACATCAAGAA

GAPDH-R GGAGTGGGTGTCGCTGTTG

### APA data analysis

Human APA cleavage sites were downloaded from PolyA_DB version 3.2 (http://exon.umdnj.edu/polya_db/v3/). PolyA_DB version 3.2 catalogs polyA sites using deep sequencing data.

### 3′ processing efficiency measurement assay

We used a bicistronic luciferase reporter construct, pPASPORT, to measure 3′ processing efficiency (39). 3′UTR of each selected gene was inserted into pPASPORT, between Renilla and Firefly luciferase genes. Plasmids were transfected into wild-type and METTL3 knockout HEK293T cells using Lipofectamine 3000 (Thermo Fisher Scientific), respectively. Renilla and Firefly luminescences were measured 24 h later using Dual-Glo Luciferase Assay System (Promega) on GloMax −96 Microplate Luminometer (Promega). All primers used to construct the reporter genes are listed in Supplementary Table S2.

### Sub-motif analysis

To obtain the expected numbers of windows with both GGACH and AAACH sub-motifs, the m^6^A sub-motif sequences were shuffled within all m^6^A peaks of a given sample. Next, the number of windows with both sub-motifs was calculated. We repeated the shuffling analysis for 10 000 times and obtained 10 000 expected numbers. To plot and compare results from different samples, we performed normalization by mean-centering the values. In brief, for a given sample, we first calculated the mean value of expected window numbers (M_expected_). Next, both observed number and the 10 000 expected numbers were divided by *M*_expected_.

### Rejected substitution score acquisition

The rejected substitution score data were from Sidow lab (http://mendel.stanford.edu/SidowLab/downloads/gerp/).

### Cross-species analysis

To conduct the CDS m^6^A conservation analysis, we used the method from a previous study (31) with some modifications. First, we required that the control A sites and the m^6^A sites were in genes with similar dN/dS values (human versus mouse). Second, we required that the control A sites and the m^6^A sites were with similar distances to the stop codon. The human-mouse pairwise alignment file was downloaded from UCSC genome browser, and human-mouse dN and dS value table was obtained via Ensembl biomart.

The proportion of m^6^A sites in the third codon that are under evolutionary constraints between human and mouse was calculated as: (the evolutionary rate at m^6^A sites (1–0.644)—the mean evolutionary rate at control sites (1 – 0.621))/(1 – 0.621).

To determine the age of individual m^6^A sites, pairwise alignment files were downloaded from UCSC genome browser.

### RNA editing site selection

Human RNA editing sites were downloaded from RADAR database (40). RADAR version 2, which includes 16 464 human RNA editing sites located at 3′UTR region, was used for analysis.

### miRNA target site selection

miRNA binding sites were downloaded from TargetScan database (Release 7.1 http://www.targetscan.org/). The TargetScan algorithm predicts biological targets of miRNAs by searching for the presence of 8mer and 7mer sites that match the seed region of each miRNA (41). A total of 669 927 and 601 858 target sites of conserved and broadly conserved miRNA families were analyzed, separately. Conserved miRNA families are conserved across most mammals, but usually not beyond placental mammals. Broadly conserved miRNA families are conserved across most vertebrates, usually to zebrafish.

### SNP data

We downloaded SNP and genotype data from the 1000 Genome Project (http://www.internationalgenome.org/). We discarded all insertion and deletion polymorphisms, SNPs with more than two alleles, SNPs monomorphic (that is, having only one allele) in all populations and SNPs that did not map uniquely to the human genome (hg19). Finally, a total of 77 664 537 SNPs were used for analysis.

### Derived allele frequency (DAF) analysis

For each SNP, we extracted the ancestral allele information from the downloaded VCF files of the 1000 Genome Project. For an RRAC motif containing an SNP, we defined it as a gain of an m^6^A site if the derived allele created an RRAC motif.

### Fst calculation

VCFtools was used to calculate Fst between populations (42).

### Haplotype homozygosity-based tests

The VCF files were obtained from 1000 Genome Project. For each m^6^A SNP, we extracted SNPs within ±2 Mb to generate individual VCF files. The Perl script vcf2impute_legend_haps.pl from impute2 (https://mathgen.stats.ox.ac.uk/impute/impute_v2.html#scripts) was used to convert a VCF file into reference panel format: one legend file and one haplotype file. The VCF files of some SNPs were failed to convert so these SNPs were excluded from the further analysis. The hap format file and map format file required by R package ‘rehh’ and selscan were formatted using the legend and haplotype files by in-house Perl scripts. iHS was calculated using the ‘ihh2ihs’ function of rehh package in R (43). XP-EHH was calculated using the‘ies2xpehh’ function of rehh package.

### Overlap with GTEx eQTL SNPs

GTEx v6p eQTL file was downloaded from GTEx website (https://gtexportal.org/home/). Only significantly associated SNPs were used.

### Overlap with GWAS data

The NHGRI-EBI Catalog of Published GWAS (44), which contains 68 741 trait-associated SNPs, was used to query for overlaps with the 102 m^6^A SNPs.

To find proxy SNPs (nearby SNPs in linkage disequilibrium) of m^6^A SNPs that are GWAS hits, we identified all SNPs in strong linkage disequilibrium with high Fst (Fst > 0.15) SNPs. This step was implemented via the VCFtools by extracting all SNPs with pairwise *r*^2^ > 0.8 based on CEU, YRI, CHB, PUR and GIH populations from the 1000 Genome Project.

### Gene ontology (GO) term analysis

GO term enrichment analysis was performed using R packages clusterProfiler (45).

## RESULTS

### m^6^A profiles across human adult tissues

**Figure 1.**
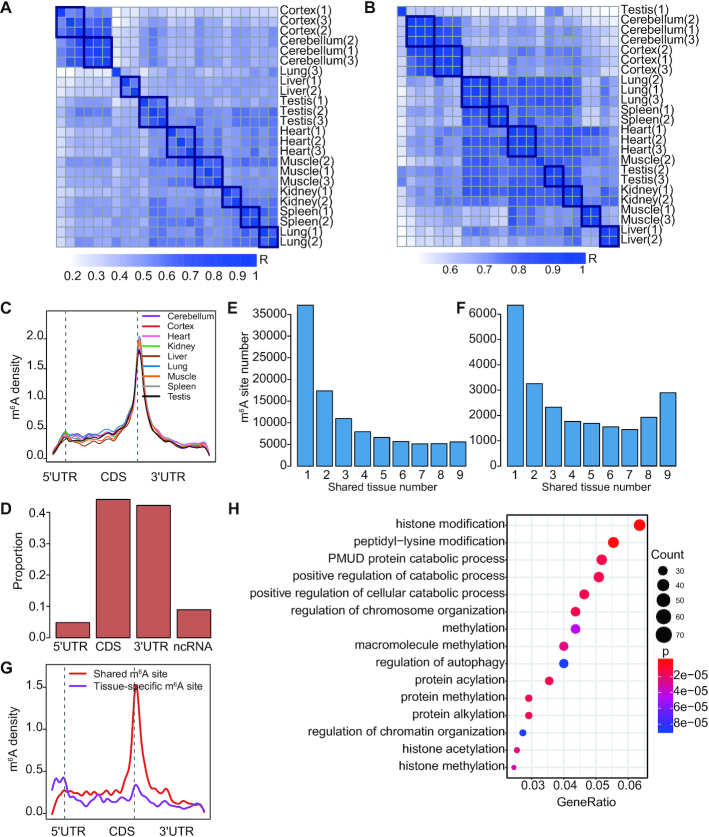
m^6^A profile in human adult tissues. (A, B) Heatmap of Pearson correlation on m^6^A peak winscores (**A**) or gene expression levels (**B**) of protein-coding genes. Gene expression levels were quantified as the number of RNA-seq reads per kilobase of transcript per million mapped reads (RPKM). (**C**) The distribution of m^6^A sites across the length of mRNA transcripts for nine adult tissues. 5′UTRs, CDSs and 3′UTRs of protein-coding genes were individually grouped into 10, 50 and 40 bins of their total length, and the percentage of m^6^A sites that fall within each bin was determined. (**D**) Genic locations of m^6^A sites of adult tissues. Sites in nine adult tissues were merged for analysis. Sites were annotated using Ensembl gene annotations and ANNOVAR software. ncRNA, Noncoding RNA. (**E**) The distribution of the numbers of m^6^A sites that are methylated in one or more tissues. (**F**) The distribution of the numbers of m^6^A sites that are methylated in one or more tissues. Only m^6^A sites within the ubiquitously expressed genes (FPKM > 3 in all tissues) were analyzed. (**G**) The distribution of tissue-specific or shared m^6^A sites across the length of mRNA transcripts for 9 adult tissues. Tissue-specific m^6^A sites, sites that are within the ubiquitously expressed genes and have a tau >0.6; shared m^6^A sites, sites that are within the ubiquitously expressed genes and have a tau <0.15. (**H**) GO terms enriched in the ubiquitously expressed genes with shared m^6^A sites (tau < 0.15). GO term analysis was performed using clusterProfiler. All ubiquitously expressed genes were used as the background. *P* values were corrected by Bonferroni adjustments and the top 15 enriched go terms were shown.

To explore the landscape of m^6^A in adult human tissues, we constructed m^6^A-seq libraries from nine human adult tissues, including cerebellum, frontal cortex, heart, kidney, liver, lung, muscle, spleen and testis. For each tissue, three individuals were profiled. m^6^A peaks were called in each sample using a winscore approach as previously described (32). m^6^A peaks found in at least 2 samples of a tissue type were considered as real m^6^A peaks. On average, we found 19 100 m^6^A peaks per tissue (Supplementary Table S3). The samples of the same tissue type clustered well according to either their m^6^A levels or gene expression levels (Figure 1A, B). Sequence logo analysis of all datasets confirmed that the called peaks are enriched in the m^6^A consensus motif RRACH (Supplementary Figure S**1**), consistent with the previous observation (2). We next inferred the m^6^A sites by searching for the RRACH motifs within the peaks (Materials and methods). As expected, m^6^A sites preferentially appeared around stop codons (Figure 1C). To evaluate the performance of the winscore peak call method we used, we applied two additional peak calling algorithms (exomePeak (36) and MeTPeak (37)) for m^6^A site call and compared m^6^A sites called from different methods. Most sites identified in our method overlapped with those identified in exomePeak or MeTPeak, and both exomePeak and MeTPeak called a lot more uniquely identified peaks (Supplementary Figure S2A-B). Compared with sites called by exomePeak or MeTPeak, as expected, sites called from our method had higher winscores (Supplementary Figure S2C). Thus, sites called using the winscore approach seemed to represent a more stringent set of m^6^A sites and were used for all following analyses. Combining all data together, we obtained a union of 101 340 m^6^A sites that span various classes of genic regions (Figure 1D, Supplementary Supplementary Table S4).

To reveal the methylation landscape across adult tissues, we examined to what extent the m^6^A sites are shared between tissues. We found that more than 36.7% of the sites were found in one specific tissue type and only 5.5% of the sites were shared in all tissues we studied (Figure 1E), thus m^6^A methylation seems to have high tissue-specificity. The profiles of four selected m^6^A peaks across tissues, as well as the m^6^A-RIP qPCR validation results, were shown in Supplementary Figure S3. To further investigate the effect of differential gene expression on the tissue-specificity of m^6^A sites, we examined m^6^A sites that were located in the ubiquitously expressed genes across human tissues. Of the 4063 ubiquitously expressed genes, 23 167 sites were identified. Of these sites, 27.4% was methylated in only one tissue type and 12.4% was shared by all tissue types (Figure 1F). These data together suggest that both tissue-specific gene expression and tissue-specific methylation contribute to the observed high tissue-specificity of m^6^A sites.

To better investigate the tissue-specificity of individual m^6^A sites, we applied the widely used tissue specificity index tau to measure the tissue-specific methylation (Materials and Methods). Tau varies from 0 to 1, where 0 means broadly expressed, and 1 is specific. Interestingly, when examining the genic locations of tissue-specific (tau > 0.6) and shared m^6^A sites (tau < 0.15), we found that shared sites tended to be located around the stop codon, while tissue-specific sites tended to be away from the stop codon (Figure 1G). This result suggests the m^6^A sites away from stop codon may perform tissue-specific functions, while m^6^A sites around stop codon are more likely to be required for the maintenance of basic cellular function, thus methylated in all cells of an organism. Consistently, the genes with shared sites were enriched in essential functions such as chromatin organization, cellular catabolic process and histone modification (Figure 1H). This tissue-specific methylation pattern was confirmed using RNA-endoribonuclease–facilitated sequencing data that identify m^6^A sites in three human tissue types (Supplementary Figure S4). Moreover, to control the potential difference in RIP efficiencies between samples, we normalized the winscores based on the top 50 peaks in each sample (Supplementary Figure S5A) and repeated the analysis. We found that the tissue-specific methylation pattern still holds (Supplementary Figure S5B). Notably, an enrichment of tissue-specific sites was found in 5′UTR (Figure 1G). To ask whether the signal in 5′UTR is m^6^Am, we performed the sequence logo analysis for tissue-specific peaks in 5′UTR regions. We found that the called peaks are enriched in the m^6^A consensus motif RRACH (Supplementary Figure S5C). Moreover, we examined the distance of the tissue-specific m^6^A sites in the 5′UTR regions to the TSS. We found that <20% of the sites are very close to TSS (Supplementary Figure S5D). These results together suggest that most tissue-specific m^6^A peaks in 5′UTRs are m^6^A, although we cannot exclude the possibility that some of the peaks that are very close to TSS are m^6^Am.

We also applied a lower or higher stringency to define m^6^A sites (i.e. we required that the sites were found in at least one sample or all samples of a tissue type) and repeated the tissue-specificity analysis. In the low stringency condition, 45.6% of the sites were methylated in only one tissue type and 4.3% of the sites were shared in all tissues; in the high stringency condition, 50.9% of the sites were methylated in only one tissue type and 0.9% of the sites were shared in all tissues. In both conditions, we consistently observed that shared sites tended to be located around the stop codon, while tissue-specific sites tended to be away from the stop codon (Supplementary Figure S6A, B). Moreover, the genes with shared sites were enriched in essential functions such as chromatin organization, cellular catabolic process and histone modification (Supplementary Figure S6C, D).

Taken together, these data reveal dynamic m^6^A methylation across tissue types, uncover both broadly or tissue-specifically methylated sites, and highlight the potentially distinct regulatory effects for m^6^A sites around and away from the stop codon.

### m^6^A methylation is enriched at non-canonical cleavage sites in 3′UTR

**Figure 2.**
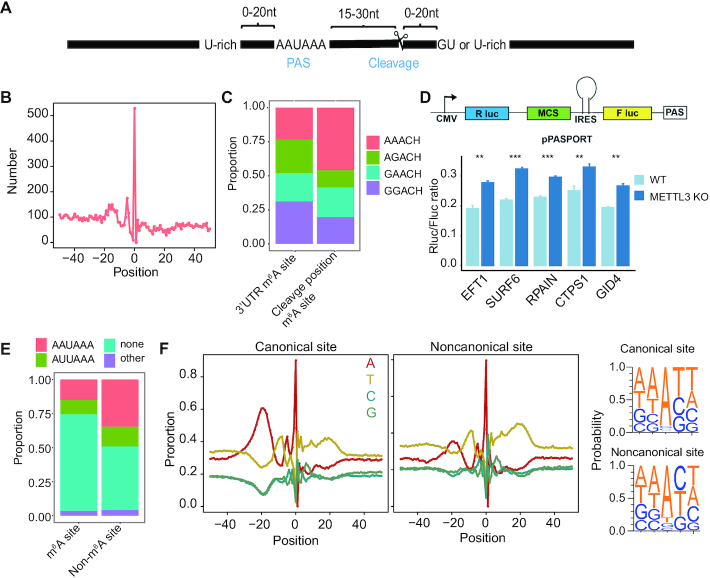
m^6^A methylation is enriched at non-canonical cleavage sites in 3′UTR. (**A**) Schematic representation of a poly(A) site and polyadenylation configuration. PAS, poly(A) signals. (**B**) The distribution of m^6^A sites around the cleavage sites. Position 0 means the cleavage position. (**C**) The proportion of m^6^A sub-motifs at the cleavage position or 3′UTR. (**D**) Top: Schematic diagram showing the bicistronic luciferase reporter system to measure 3′ processing efficiency. Fragment of interest was inserted into the MCS position (i.e. between Renilla (R luc) and Firefly luciferase (F luc) genes). Efficient mRNA 3′ processing at the tested PAS leads to high expression of Renilla luciferase gene and low expression of Firefly luciferase gene, while poor mRNA 3′ processing results in the opposite mode of gene expression. Therefore, the Renilla/Firefly ratio provides a quantitative measurement of the processing efficiency at the tested PAS. Bottom: Luciferase reporter assays to determine the relative PAS activity of 5 selected sites in wild-type and METTL3 knockout cells. Four biological replicates were performed and statistical significance was calculated using Student's t-test. The relative PAS activities are represented as mean ± sd. **P < 0.01; ***P < 0.001. (**E**) The proportion of canonical and noncanonical PAS for m^6^A and non-m^6^A cleavage sites. Canonical groups: AAUAAA, AUUAAA and other; noncanonical group, none. (**F**) Left: nucleotide sequence composition around all 3P-seq–identified canonical and noncanonical poly(A) sites. Position 0 means the cleavage position. Cleavage sites located in 3′UTR or 3′UTR downstream 1kb regions were analyzed. Right: The sequence context 2nt upstream and downstream of the cleavage position.

Polyadenylation processing of pre-mRNAs is an essential step in the generation of mature mRNAs. It includes an endonucleolytic cleavage followed by polyadenylation (46). A cleavage site is typically located in the downstream 15–30nt of the poly(A) signal (PAS) (Figure 2A). Most eukaryotic genes harbor multiple PASs, leading to expression of alternative polyadenylation (APA) isoforms. m^6^A is known to be associated with APA selection (30,47,48), however, whether m^6^A methylation directly regulates polyadenylation is unknown. The generation of the comprehensive list of m^6^A sites, along with the map of poly(A) cleavage sites in human (49), enables us to systematically examine the relationship between m^6^A methylation and cleavage. To do so, we first examined the distribution of the distance between a cleavage site and the nearest m^6^A site. Unexpectedly, we found that m^6^A sites are highly enriched in the cleavage site position (Figure 2B). This observation was confirmed using miCLIP and RNA-endoribonuclease–facilitated sequencing data that identify m^6^A sites with single-nucleotide-resolution (Supplementary Figure S7A). In addition, compared with 3′UTR m^6^A sites that were not located at the cleavage position, m^6^A sites at the cleavage position tended to be enriched in AAACH sub-motif (Figure 2C).

To examine the effect of m^6^A on PAS regulation, we utilized a PAS reporter assay to measure the impact of m^6^A on poly(A) site processing efficiency (Figure 2D). We first generated METTL3 knockout HEK293T cells (Supplementary Figure S7B, C) and confirmed the reduced m^6^A level (Supplementary Figure S7D). Next, we selected five m^6^A sites that were in the cleavage site position and methylated in HEK293T cells (Supplementary Figure S7E). We subcloned ∼ 300 bp region around each m^6^A sites into the reporter plasmid and transfected each of the reporters into both wild-type and METTL3 knockout cells. We found that these m^6^A located PAS regions had a higher processing efficiency in the METTL3 knockout cells (Figure 2D). This result suggests that m^6^A may repress polyadenylation processing.

APA events can lead to the production of noncanonical mRNA isoforms, affecting the fate of the transcript and the nature of the products of translation (50). To ask if m^6^A may be involved in such regulation, we divided APA sites into canonical and noncanonical groups and examined their association with m^6^A. Interestingly, we found that m^6^A sites tended to be located at the noncanonical APA sites (Figure 2E). An examination of the nucleotide compositions around the cleavage sites revealed that, compared with canonical APA sites, noncanonical APA sites had an enrichment of Cs at the immediate downstream of the cleavage position (Figure 2F), thus are more likely to form the RRACH motif required for m^6^A methylation.

Taken together, we unexpectedly observed an enrichment of m^6^A sites in the cleavage position, particularly for the noncanonical APA events. This observation raises the possibility that the methylation status of the cleavage position may affect the cleavage efficiency directly, therefore regulating APA selection. Consequently, the dynamic methylation of m^6^A at cleavage position across tissue types may contribute to the dynamic regulation of APA across tissue types.

### Developmental dynamics of m^6^A methylation

**Figure 3.**
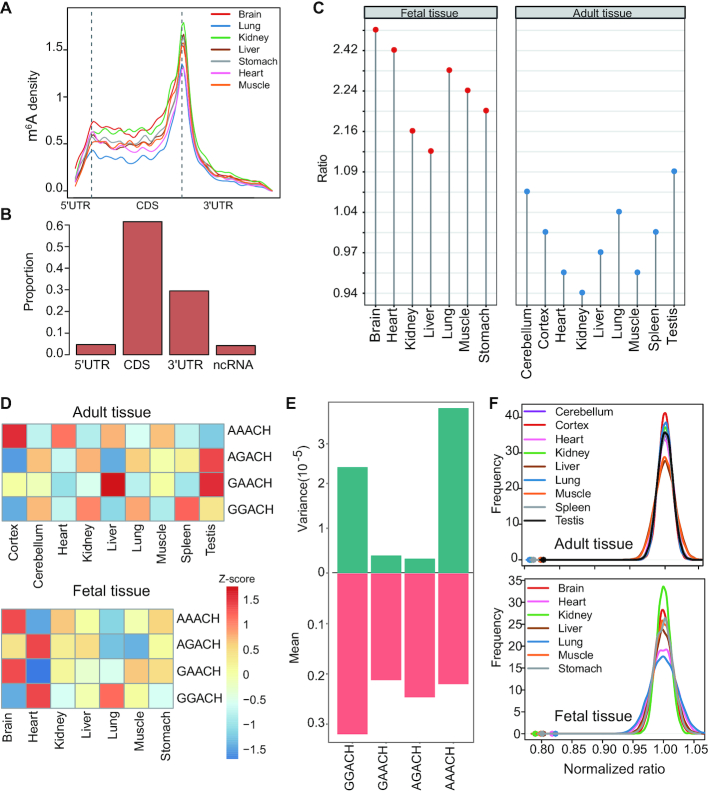
Developmental dynamics of m^6^A profile. (**A**) The distribution of m^6^A sites across the length of mRNA transcripts for 7 fetal tissues. (**B**) Genic locations of m^6^A sites of fetal tissues. Sites in seven fetal tissues were merged for analysis. (**C**) The ratio between the CDS m^6^A site number and 3′UTR m^6^A site number in fetal and adult tissues. (**D**) Heatmap showing the normalized proportion (the values were centered and scaled in the row direction) of 4 m^6^A sub-motifs in adult and fetal tissues. (**E**) Variance and mean value of the proportion of 4 sub-motifs across human adult tissues. (**F**) GGACH and AAACH sub-motifs tend to locate in different windows for all tissue types we studied. Color dots indicate the observed numbers of m^6^A peaks with both GGACH and AAACH sub-motifs. The distribution of the expected numbers was generated by the shuffled data. P is the fraction of the distribution on the left side of the dots. It is found that the observed numbers of m^6^A peaks with both GGACH and AAACH sub-motifs are significantly smaller than that of the shuffled data (*P* < 0.0001). The x-axis is the ratio of the number of windows with both sub-motifs over the mean number of windows with both sub-motifs calculated using the shuffled data.

To understand the developmental dynamics of m^6^A methylation, we compared the m^6^A profile between fetal and adult tissues. m^6^A profiles of seven human fetal tissues, including brain, heart, kidney, liver, lung, muscle and stomach, were used for analysis (23). Among these tissues, 5 tissue types were in common between fetal and adult samples. m^6^A peaks and sites of fetal tissues were called as we did in our adult tissue data. We confirmed that the samples of the same tissue type clustered together according to either their m^6^A levels or gene expression levels (Supplementary Figure S8A, B). In addition, the called peaks were enriched in the m^6^A consensus motif RRACH (Supplementary Figure S8C). In total, we obtained a union of 60 440 fetal m^6^A sites. These fetal m^6^A sites also preferentially appeared around stop codons (Figure 3A) and spanned various classes of genic regions (Figure 3B). Interestingly, fetal and adult tissues showed distinct distributions of m^6^A sites along the transcripts. Although m^6^A sites preferentially appeared around stop codons in both fetal and adult tissues, the CDS regions of fetal tissues showed clearly higher m^6^A proportions than that of adult tissues (Figure 3C). Because the m^6^A profile data of fetal tissues were generated using a different RIP procedure and antibody from our method, to exclude the possibility that the observed difference is due to technical issue, we performed two analyses. First, we constructed m^6^A-seq libraries using the same RNA sample with two different methods. We found that sites called from both methods had the same distribution across the transcripts (Supplementary Figure S9A), suggesting that the use of different library construction methods had no significant impact on m^6^A distribution analysis. Second, we analyzed an independent fetal tissue m^6^A-seq data, which include 3 post-conception week 11 (PCW11) fetal human brain samples, 3 mouse developing brain (E13.5) samples and 2 human 47 day forebrain organoid samples (51). These m^6^A-seq data were generated with the same RIP procedure as our data. The enrichment of CDS sites in fetal tissues was confirmed in this data set (Supplementary Figure S9B). VIRMA is known to mediate preferential m^6^A mRNA methylation in 3′UTR and near stop codon (48). An examination of VIRMA expression revealed that, compared with adult tissues, fetal tissues had higher expression levels (Supplementary Figure S9C, D), thus the observed difference between fetal and adult tissues may be due to other unknown regulators.

### The regulation of m^6^A motifs across tissue types or developmental stages

We noted that although the m^6^A consensus motif RRACH is enriched in all tissue types examined, the detailed motifs vary between tissues (Supplementary Figure S1 and Supplementary Figure S8C). To examine the motif dynamics and regulation between tissues, we divided the RRACH motif into four sub-motifs (GGACH, AGACH, GAACH and AAACH) for analysis. We found that different tissues had different sub-motif preferences (Figure 3D). For example, AAACH was overrepresented in both fetal brain and adult frontal cortex. Interestingly, we found that the proportions of AAACH and GGACH sub-motifs were most variable between tissues, while the proportion of AGACH and GAACH sub-motifs were consistent across tissues (Figure 3E and Supplementary Figure S10A). Moreover, during development, the sub-motifs of some tissues were changing but others were not (Supplementary Figure S10B).

m^6^A is installed by a multicomponent methyltransferase complex. Besides the core methyltransferase subunits, it also contains other proteins that interact with core subunits to methylate specific positions (52). The combination of core methyltransferase subunits with different interacting proteins may lead to different motif preference. The observation above suggests that GGACH and AAACH sub-motifs may be installed by core methyltransferase subunits with distinct interacting proteins. If so, we expect that these two sub-motifs may tend to occur in different peak regions. To ask if this is true, we shuffled the m^6^A sub-motif sequence within the peaks and calculated the numbers of peaks with both AAACH and GGACH sub-motifs (Materials and methods). We found that the observed number of peaks with both AAACH and GGACH sub-motifs were significantly less than the expected numbers in all tissues we examined (Figure 3F), consistent with our expectation.

### The evolutionary landscape of human m^6^A methylation

Having revealed the dynamics of m^6^A methylation across human tissues, we next investigated its evolution. First, we examined the cross-species conservation of m^6^A sites to assess the strength of selective pressure on individual m^6^A sites.

**Figure 4.**
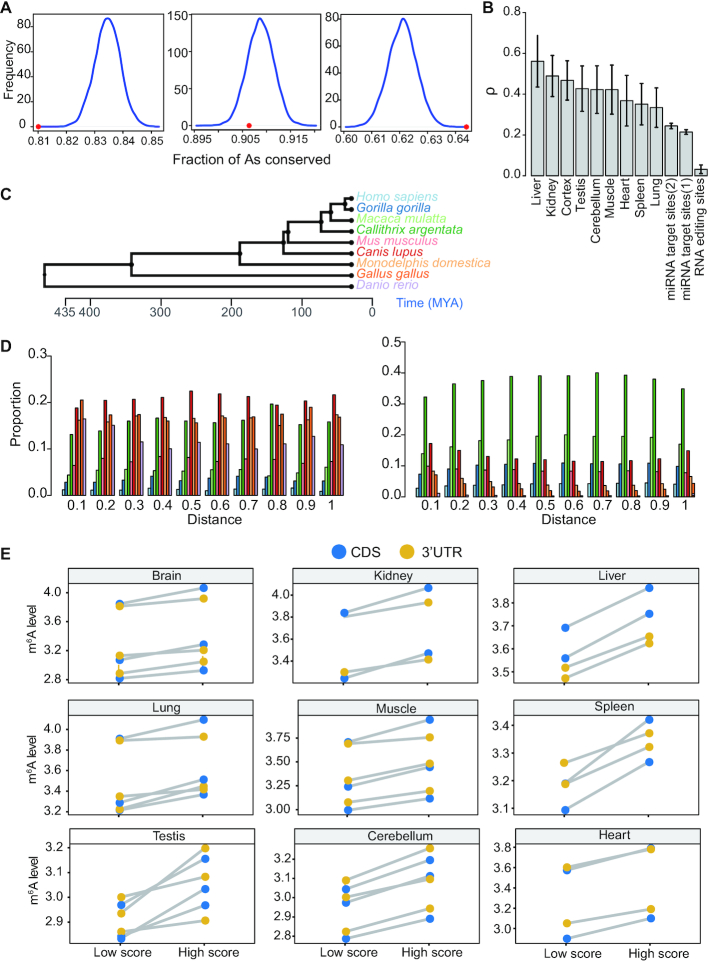
Natural selection on m^6^A inferred by cross-species analysis. (**A**) Comparison of evolutionary conservation between m^6^A and control A sites (non- m^6^A RRACH). Frequency distributions of the fraction of conserved control sites in 10,000 random sets with the sample size equal to the number of m^6^A sites at three codon positions were plotted separately. Red dots indicate the fraction of conserved m^6^A sites. P is the fraction of the distribution on the right side of the dots. First codon, *P* = 1; second codon position, *P* = 0.89; third codon position, *P* <0.0001. (**B**) Estimates of ρ for m^6^A RRACH motifs, A-to-I RNA editing triplet motif and miRNA binding sites. ρ represents the fraction of sites under selection within functional elements, which is calculated by INSIGHT. m^6^A sites, m^6^A RRACH motifs in 3′UTR region; RNA editing sites, nonrepetitive A-to-I RNA editing sites in 3′UTR region; miRNA target sites, 3′UTR miRNA binding sites for conserved (1) or broadly conserved (2) miRNA families. The regions that match the seed region of the miRNAs were used for analysis. (**C**) A tree representing the schematic phylogeny of the species studied. (**D**) The age distribution of m^6^A sites (left) and control non-m^6^A RRACH sites (right) across the 3′UTR region. m^6^A sites and control sites were grouped into 10 bins based on their distance to stop codon. The age of a site was based on the most distantly related species in which the site was conserved. The color codes for the age are the same as in (C). (**E**) Comparison of methylation levels between m^6^A sites under strong and weak constraints in CDS and 3′UTR. m^6^A sites with top 25% (High score, strong constraint) and bottom 25% (Low score, weak constraint) rejection scores were compared. m^6^A peak winscore of a site was used to represent m^6^A level. Each dot represents the median methylation level of the CDS or 3′UTR sites; biological replicates of each tissue type were plotted, separately.

For CDS sites, we examined the selection pressure of m^6^A sites in different codon positions, as they may be subject to different evolutionary constraints. To estimate the strength of selection pressure, we chose to compare the fraction of conserved m^6^A sites between human and mouse with that of the control A sites, as previously described (31). It is known that different genes are subject to different selection pressure. To control such effect, for control A sites in non-m^6^A RRACH motifs, we selected sites in genes with similar selection pressure, i.e. similar dN/dS ratio, as the m^6^A sites. In addition, m^6^A sites tended to be located in the 3′end of the CDS region. An examination of evolutionary constraint across the CDS region revealed that As in different CDS regions are subject to different levels of evolutionary constraint (Supplementary Figure S11). To control such effect, for each gene, we grouped the CDS regions into 20 bins and required that the control A sites were located in the same bin as the m^6^A sites. We found that m^6^A sites in different codon positions had different conservation patterns, consistent with a previous study (31). The m^6^A sites in the first codon position were less conserved than control A sites, while m^6^A sites in the third codon were much conserved than control A sites (Figure 4A). We estimated that 6% of the m^6^A sites in the third codon are under evolutionary constraints (Materials and methods), therefore likely functional. m^6^A methylation is known as a barrier to tRNA accommodation and translation elongation, and m^6^A in the first codon position has the strongest effect on delaying tRNA accommodation (53). Therefore, the effect of m^6^A modification in the first codon position may be generally detrimental, and more likely to be less conserved.

For 3′UTR sites, we used INSIGHT (Inference of Natural Selection from Interspersed Genomically coHerent elemenTs) (54), a method for measuring the influence of natural selection for short, widely scattered noncoding elements, to estimate the proportion of m^6^A sites that are under negative selection (in other words, are functional). INSIGHT obtains information about natural selection by contrasting patterns of polymorphism and divergence in m^6^A motifs (RRACH) with those in flanking neutral regions. We obtained estimate of ρ that range from 0.33 to 0.56 for different tissues (Figure 4B). As a comparison, we examined two additional classes of regulatory elements in 3′UTR region: A-to-I RNA editing sites and miRNA binding sites. For RNA editing sites, we examined the editing site triplet motif (11,55) and estimated ρ = 0.03 (Figure 4B). For miRNA binding sites identified using Targetscan algorithm (41), we obtained an average estimate of ρ = 0.21 and 0.24 for conserved and broadly conserved miRNA families, respectively (Figure 4B). Thus, unexpectedly, we detected a much stronger signature of natural selection in m^6^A motifs compared with other post-transcriptional regulatory elements, with about half of the nucleotides estimated to be under negative selection. Next, we ask if the selection pressure on 3′UTR m^6^A sites is associated with their locations. We grouped m^6^A sites into 10 bins based on their locations and used phylostratigraphy data (Figure 4C) to examine the age of human m^6^A sites in each bin. We found that m^6^A sites had an older age than the control sites in all bins, suggesting that 3′UTR m^6^A sites are generally subject to negative selection (Figure 4D).

Second, we examined the relationship between m^6^A level and m^6^A site conservation. We obtained the rejected substitution scores, a score to measure the nucleotide-level constraint (56), of all m^6^A sites, and compared the methylation levels between m^6^A sites under stronger constraints and weaker constraints. Interestingly, we found that m^6^A sites under stronger constraints had higher methylation levels (Figure 4E). This observation suggests that the conserved m^6^A sites are optimized for m^6^A writer binding and methylation, thus likely functional.

### Positive selection of m^6^A sites inferred from population genomic analysis

**Figure 5.**
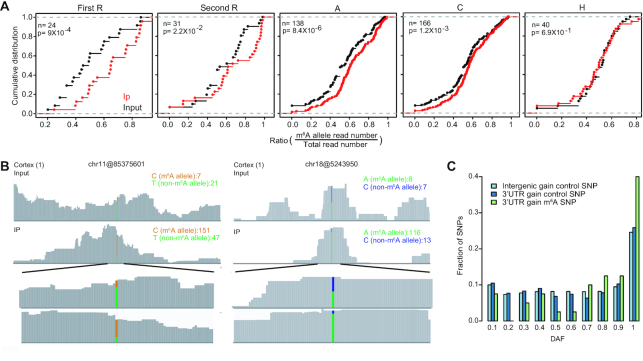
Positive selection inferred from SNP genotype data. (**A**) Comparison of the m^6^A allele ratio between IP and input samples of heterozygote m^6^A SNPs. Only heterozygote SNPs with one genotype matching the RRACH motif and the other genotype not matching the RRACH motif were considered. SNPs located in each position of RRACH motifs were analyzed, separately. *P* values were calculated with the Kolmogorov-Smirnov test. (**B**) Examples of m^6^A peaks with SNPs. For chr11@85375601 m^6^A site, the C allele (GAA*C*T, m^6^A site is underlined and the SNP is marked in italics) matches the m^6^A motif and the T allele (GAA*T*T) disrupts the motif. For chr18@5243950 m^6^A site, the A allele (A*A*ACA) matches the m^6^A motif and the C allele (A*C*ACA) disrupts the motif. For both sites, compared with the non-m^6^A allele, the m^6^A allele is highly enriched in the IP sample. (**C**) DAF distributions of 1,000 Genome Project SNPs of m^6^A RRAC motifs for 3′UTR sites. The derived alleles that create m^6^A motifs were analyzed. Two groups of control sites were selected: (1) ‘RRAC’ from all non-m^6^A RRACH motifs located at 3′UTR region; (2) ‘RRAC’ from all RRACH motifs in the intergenic region. *P* values were calculated with a one-sided Mann-Whitney U test comparing the DAF distribution of SNPs in m^6^A RRAC motif with the distribution of SNPs in control sites. 3′UTR control group versus m^6^A group, *P* = 0.00037; intergenic group versus m^6^A group, *P* = 0.000013.

To ask if the m^6^A sites that were recently gained during human evolution were under positive selection, we analyzed SNP genotype data from the 1,000 Genome Project (Materials and methods). To prevent the ascertainment bias between functional classes, 5′UTR, 3′UTR of protein-coding genes and ncRNAs were analyzed. CDS region was excluded from this analysis, because it is difficult to distinguish if the selection signals are from m^6^A methylation or other factors unrelated to m^6^A methylation, such as amino acid changes. First, we asked if SNPs located within the RRACH motifs do affect methylation. To do so, we first identified all heterogeneous m^6^A SNPs in 27 adult tissue samples using the input RNA-seq data. Next, we calculated the m^6^A allele ratio (m^6^A allele read number/ total read number) using reads covered the selected SNPs for both IP and input samples. Last, we compared the m^6^A allele ratios between IP and input samples and each position of the RRACH motif was examined separately. We found that m^6^A allele had higher ratios in IP samples for SNPs located in the RRAC positions (Figure 5A). Thus SNPs located at the RRAC positions but not the H position affect methylation status. The read coverages of two representative heterogeneous m^6^A SNPs in IP and input samples were shown in Figure 5B. Next, we examined the DAF spectrums for a derived allele that are located in the RRAC positions and create an m^6^A motif. Different DAF distributions were directly compared using a Mann-Whitney test, as previously described (27). Interestingly, the DAF distribution for the SNPs of which the derived allele creates m^6^A motifs was significantly skewed toward high-frequency alleles relative to matched control or neutral sites, suggesting that some of these SNPs are subject to positive selection (Figure 5C).

The excess of high-frequency derived alleles that create m^6^A motifs promotes us to further characterize the m^6^A site SNPs that are likely under positive selection. To have a comprehensive scan of positively selected SNPs related to m^6^A modification, we examined not only the SNPs of which the derived allele is m^6^A allele but also those of which the ancestral allele is m^6^A allele. Because it is plausible that selection may sometimes switch to favor an ancestral allele that has been segregating in the population. We first identified m^6^A SNPs that have been highly differentiated among populations (measured by the Fst parameter) and then determined where these differentiation events might be driven by positive selection during human evolution. In total, we identified 102 highly differentiated SNPs (Fst > 0.15) (Supplementary Table S5). Of these SNPs, 37 showed evidence of selection in iHS (the integrated Haplotype Scores) (57) and/or XP-EHH (Cross Population Extended Haplotype Homozogysity) (58) tests (Supplementary Tables S6–S7).

Among these differentiated SNPs, 81 SNPs are located in protein-coding genes. The remaining 21 SNPs are located in ncRNA genes, suggesting that m^6^A sites in ncRNA genes may be another class of targets of recent positive selection. The protein-coding gene list contains a number of genes involved in biological pathways thought to be recently targeted by positive selection, such as metabolism of carbohydrates, lipids and brain development (57). Particularly, it includes 25 genes that have previously been characterized as positively selected genes in the human lineage or across human populations (Supplementary Table S5).

As m^6^A is known to affect mRNA stability, to further understand the potential regulatory effects of these SNPs, we examined the overlaps between these variants and eQTL SNPs from the Genotype-Tissue Expression (GTEx) project. Of the 102 SNPs, 60 are associated with gene expression abundance (Supplementary Table S8), suggesting that these m^6^A SNPs may contribute to the expression changes. Last, we characterized the potential phenotypic effect of these variants. Using Genome-wide association study (GWAS) data, we found that a substantial proportion of the 102 m^6^A SNPs is disease- or trait- associated SNPs (Supplementary Table S8), such as those associated with HDL cholesterol levels, body mass index, and atherosclerosis.

## DISCUSSION

The importance of m^6^A as a post-transcriptional modification has been appreciated, but the evolution, function and regulation of individual m^6^A sites remain largely unknown, in part because of insufficient data about its prevalence and dynamics. Here, we compiled the m^6^A methylomes of major adult human tissues, providing a valuable resource for future studies of the regulation and functions of this modification. We reveal that the distribution and motifs of m^6^A vary across tissue types or during development, suggesting that m^6^A is widely regulated by trans factors and involved in human development. Notably, it is known that the variation of postmortem conditions in different samples may affect RNA integrity and gene expression quantification. Since it is unknown that whether m^6^A in post-mortem tissues represents the in-situ state and whether m^6^A is more unstable than RNA itself, caution needs to be taken when using the m^6^A maps generated with postmortem tissues.

We used comparative genomics and population genetics approaches to show that a significant negative selection has acted on m^6^A sites, particularly the ones in the third codon position and 3′UTR. Current opinion on m^6^A modification believes that despite the functional importance of this modification, the single m^6^A site seems dispensable, as long as a transcript is methylated and can be recognized by a member of the major m^6^A reader YTH family (20,59,60). Our data, however, do not support this view and suggest that many of the single sites should be functionally important, most likely because their functions are position-dependent. Furthermore, with 1,000 Genome Project data, we identified a number of m^6^A SNPs whose patterns of allelic variation are not consistent with neutrality. There is a functional difference between alleles and finally the functional difference would result in a phenotypic effect that would be influenced by selection. These SNPs are enriched in the genes related to the immune system, dietary fatty acid processing and neuronal functions, consistent with the recently identified functions of m^6^A modification (16,61–64).

In summary, our work provides a resource of m^6^A profile in humans for future studies of m^6^A regulation and functions. Furthermore, our data provide independent evidence for the functional importance of m^6^A modification from the evolutionary perspective, and also suggests an unexpected role of m^6^A modification in recent human evolutionary adaptation and disease susceptibility.

## DATA AVAILABILITY

The sequence data have been deposited in the NCBI GEO database under the accession code GSE122744.

## Supplementary Material

gkaa347_Supplemental_Files
