# Supplementary material for: Dynamic landscape and evolution of m6A methylation in human
Source: Nucleic Acids Res. 2020 May 14;48(11):6251–64. doi: 10.1093/nar/gkaa347 (PMC7293016; doi:10.1093/nar/gkaa347)
Supplement: gkaa347_Supplemental_Files [file gkaa347_supplemental_files.zip › Zhang Supplementary_information_revision2.pdf]

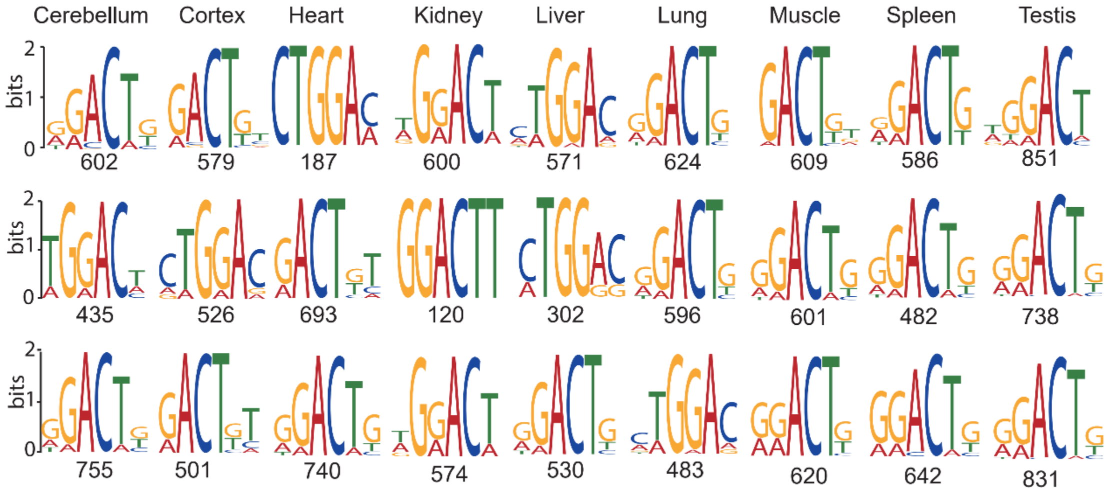

Figure S1. The MEME-deduced consensus motif.

The top MEME-deduced consensus motif for the 1,000 best-scoring m^{6}A peaks identified the canonical m^{6}A motif RRACH in all adult tissues we profiled. At a given position, the height of a nucleotide reflects its frequency. The numbers of sites contributing to the construction of the motif are also indicated.

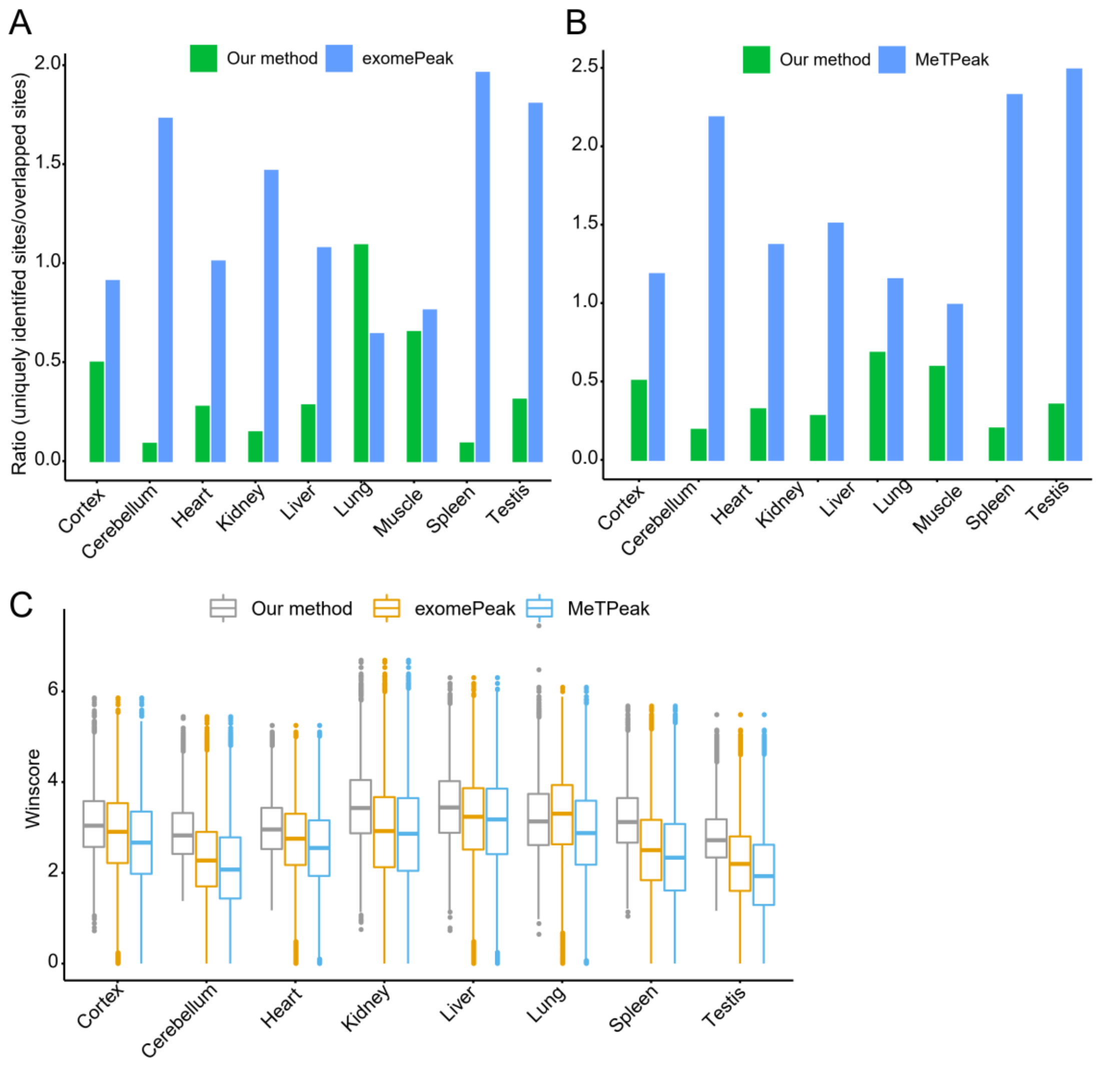

Figure S2. Comparison of different m^{6}A call approaches.

(**A-B**) Comparison of our m^{6}A sites with exomePeak sites (**A**) and with MeTPeak sites (**B**).

(**C**) Boxplot showing the winscores for peaks called using different approaches. For each tissue type, peaks called from replicates were combined together for analysis.

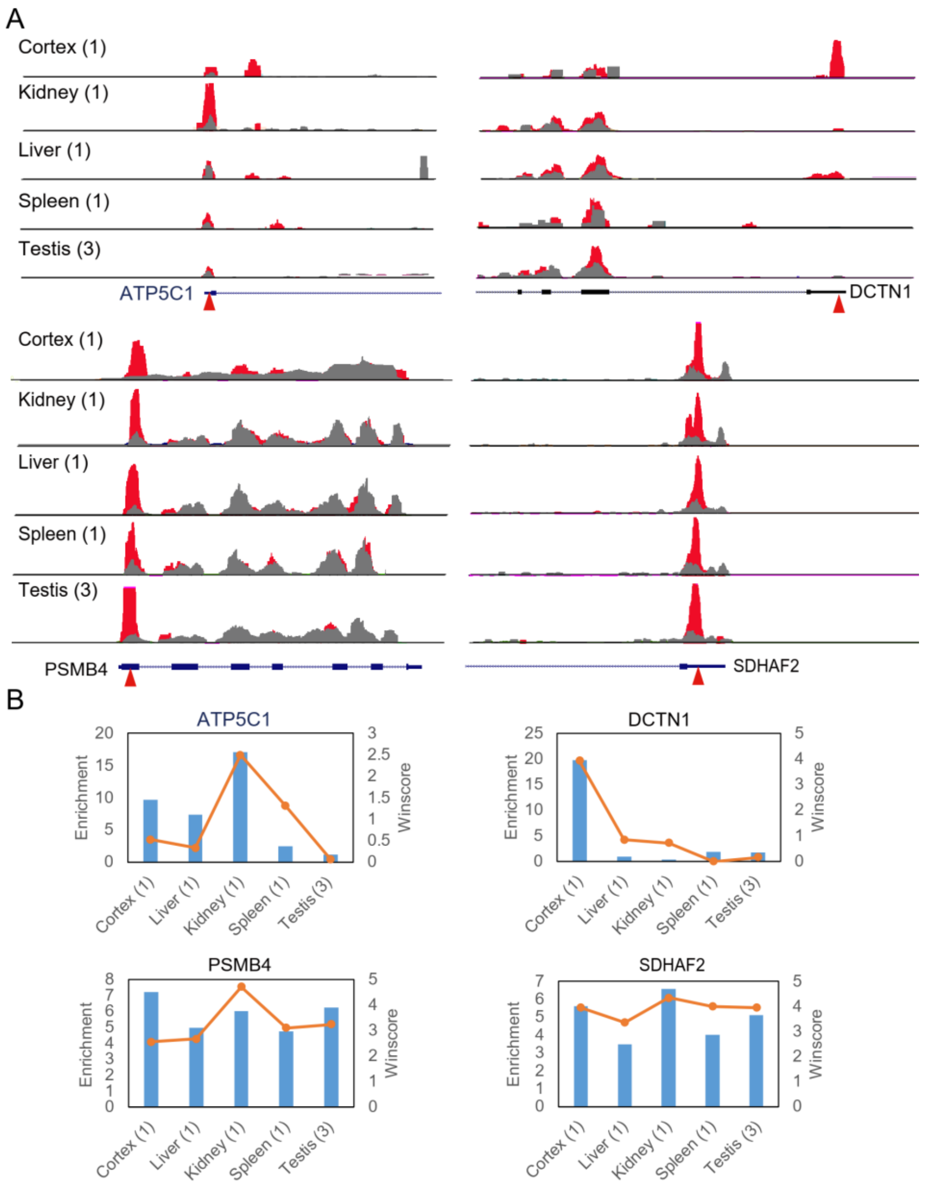

Figure S3. The profile of 4 selected m^{6}A sites across tissues.

(**A**) IGV browser view of the read coverage of genes with 4 selected m^{6}A sites. m^{6}A-seq data in five tissues are shown. m^{6}A positions are marked by the red triangle. IP sample, red; input sample, gray.

(**B**) m^{6}A-RIP qPCR results for the selected sites in 5 tissues. Data are represented as mean of two technical replicates. Bars represent the m6A-RIP qPCR enrichment results. Lines represent the corresponding winscore results.

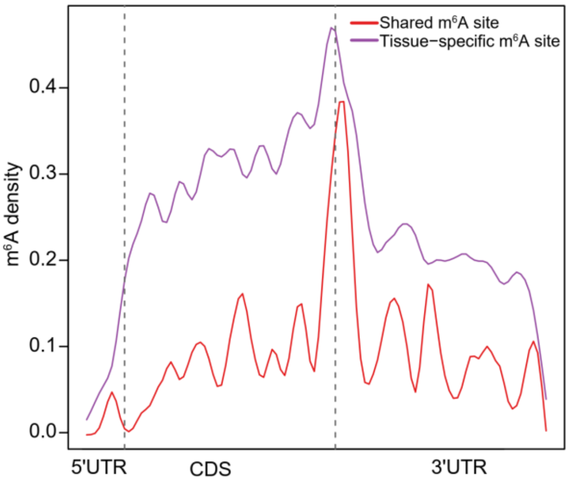

Figure S4. The distribution of tissue-specific or shared m^{6}A sites across mRNA transcripts.

m^{6}A sites that were identified with RNA-endoribonuclease–facilitated sequencing method in three human tissues (brain, liver and kidney) were used for analysis (1)**.** Tissue-specific m^{6}A sites, sites that are identified in only one tissue type; shared m^{6}A sites, sites that are identified in all three tissue types.

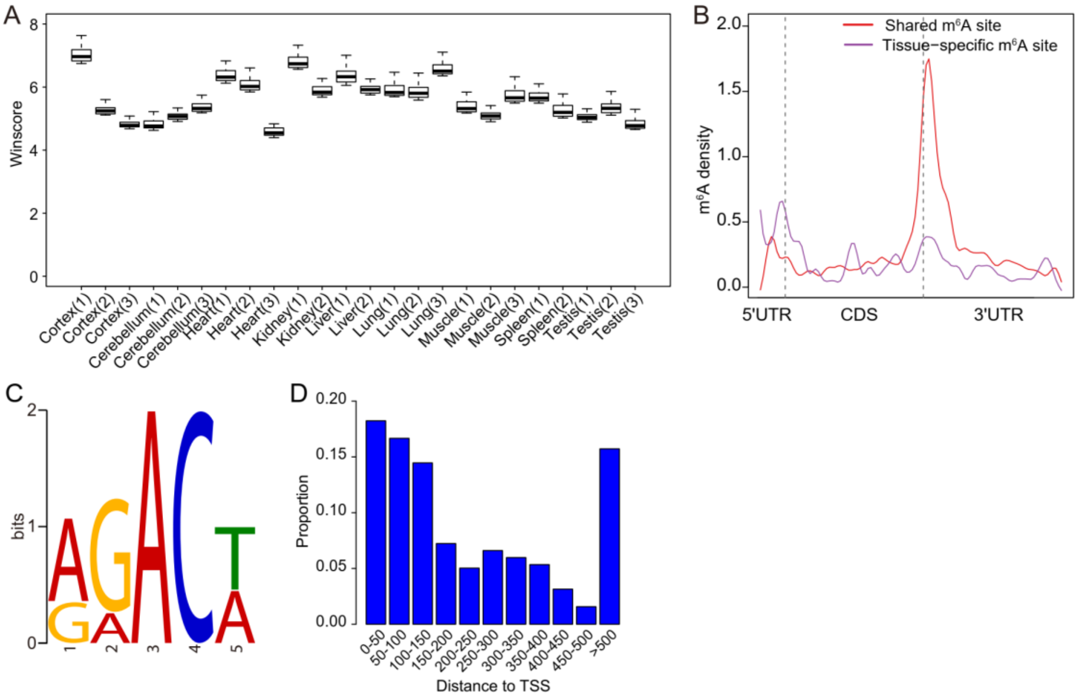

Figure S5. Tissue-specific m^{6}A methylation analysis.

(**A**) Boxplot showing the winscores of top 50 m^{6}A peaks in each sample.

(**B**) The distribution of tissue-specific or shared m^{6}A sites across the length of mRNA transcripts for 9 adult tissues. To normalize the potential difference of IP efficiency of different samples, we assumed that the top 50 m^{6}A peaks in each sample had similar m^{6}A levels (close to 1). Based on this assumption, we first calculated the median winscore value (M_{value}) of the top 50 m^{6}A peaks of a sample. Next, the winscore values of all m^{6}A peaks in this sample were divided by M_{value} for normalization. Finally, tissue-specific or shared sites were calculated based on the normalized winscores. Tissue-specific m^{6}A sites, sites that are within the ubiquitously expressed genes and have a tau > 0.6; shared m^{6}A sites, sites that are within the ubiquitously expressed genes and have a tau < 0.15.

(**C**) The top MEME-deduced consensus motif for the tissue-specific m^{6}A peaks identified the canonical m^{6}A motif RRACH.

(**D**) The proportions of tissue-specific m^{6}A sites near the TSS.

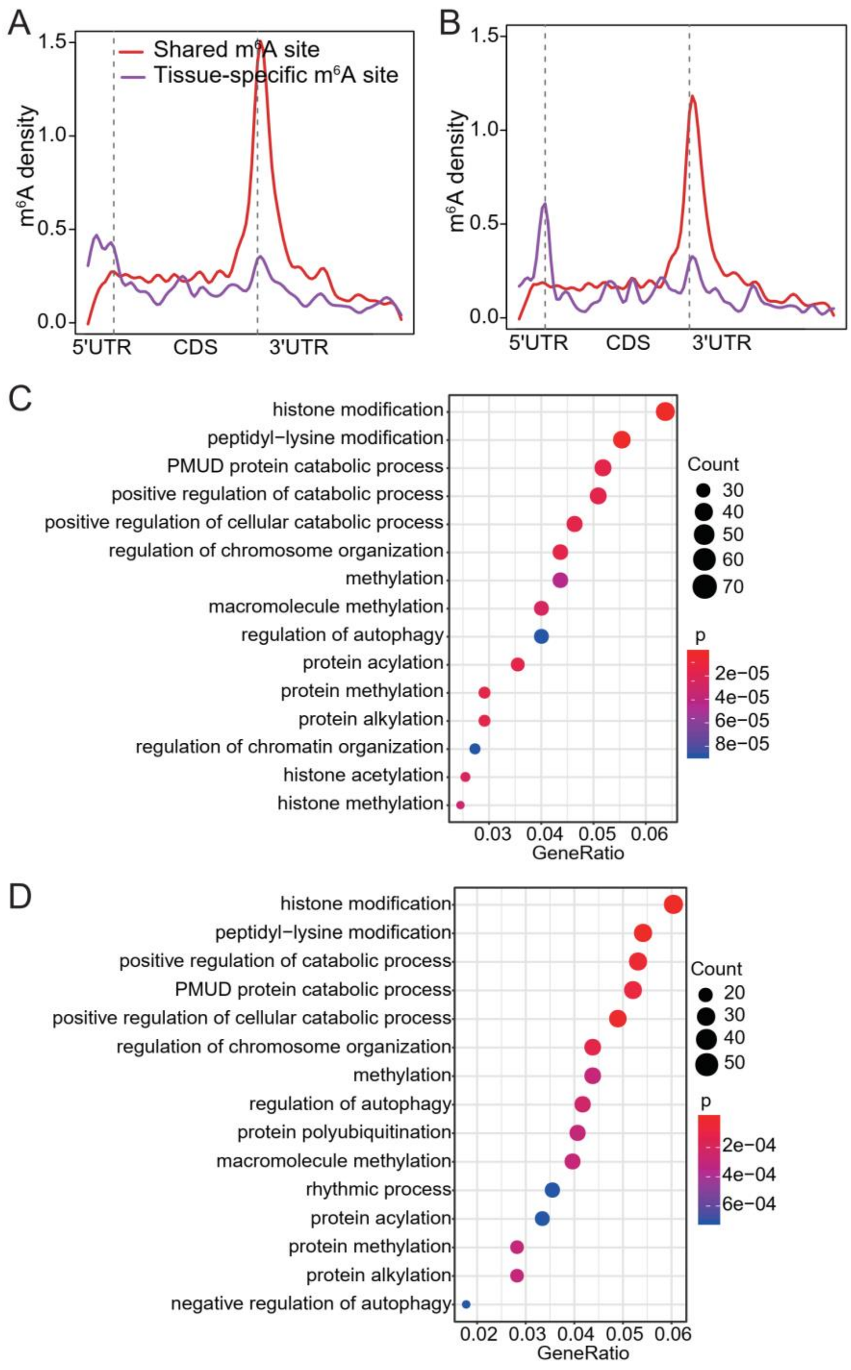

Figure S6. Tissue-specificity analysis based on low- and high- stringency m^{6}A sites.

(**A-B**) The distribution of tissue-specific or shared m^{6}A sites across the length of mRNA transcripts for 9 adult tissues. Tissue-specific m^{6}A sites, sites that are within the ubiquitously expressed genes and have a tau > 0.6; shared m^{6}A sites, sites that are within the ubiquitously expressed genes and have a tau < 0.15. Low stringency (**A**)

and high stringency (**B**) sites were analyzed, respectively.

(**C-D**) GO terms enriched in the ubiquitously expressed genes with shared m^6A sites (tau < 0.15). Low stringency (**C**) and high stringency (**D**) sites were analyzed, respectively. GO term analysis was performed using clusterProfiler. All ubiquitously expressed genes were used as the background. P values were corrected by Bonferroni adjustments and the top 15 enriched go terms were shown.

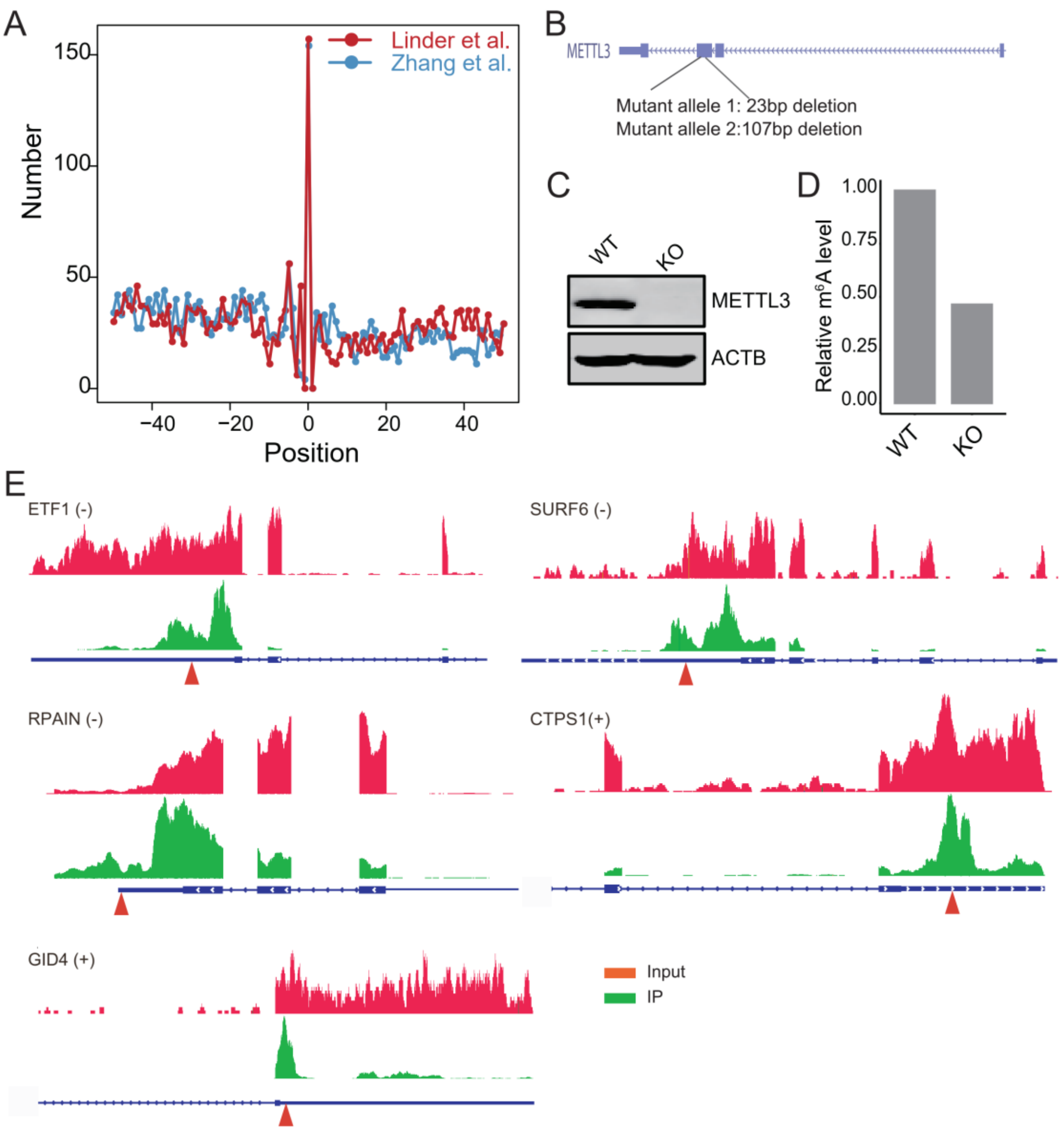

Figure S7. The distribution of m^{6}A sites around the cleavage sites.

(**A**) Linder et al. and Zhang et al., m^{6}A sites with single-nucleotide-resolution identified with miCLIP method (2) or RNA-endoribonuclease–facilitated sequencing method (1)**.** Position 0 means the cleavage position.

(**B**) Schematic representation of METTL3 knockout generation using the CRISPR-Cas9 system.

(**C**) Western blot validation of METTL3 mutagenesis.

(**D**) Overall m^{6}A quantification for poly (A) mRNAs from wild-type and METTL3 knockout cells using EpiQuik m^{6}A RNA Methylation Quantification Kit.

(**E**) IGV browser view of the read coverage of genes with m^{6}A sites selected for PAS luciferase reporter assay. m^{6}A-seq data in HEK293T cells are shown. m^{6}A positions are marked by the red triangle.

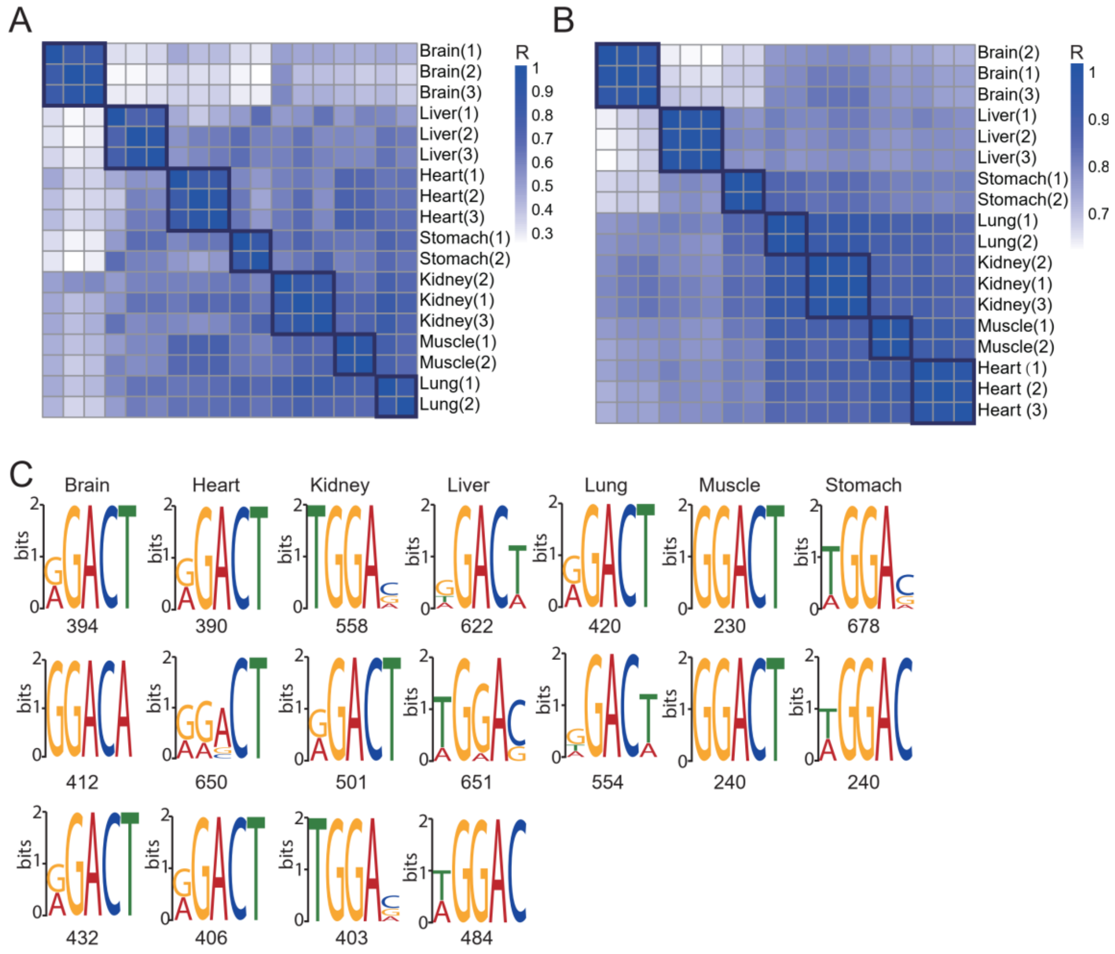

Figure S8. m^{6}A profile in human fetal tissues.

(**A-B**) Heatmap of Pearson correlation on m^{6}A peak winscores (**A**) or gene expression levels (**B**) of protein-coding genes. Gene expression levels were quantified as RPKM. (**C**) The top MEME-deduced consensus motif for the 1,000 best-scoring m^{6}A peaks in fetal tissues. At a given position, the height of a nucleotide reflects its frequency. The numbers of sites contributing to the construction of the motif are also indicated.

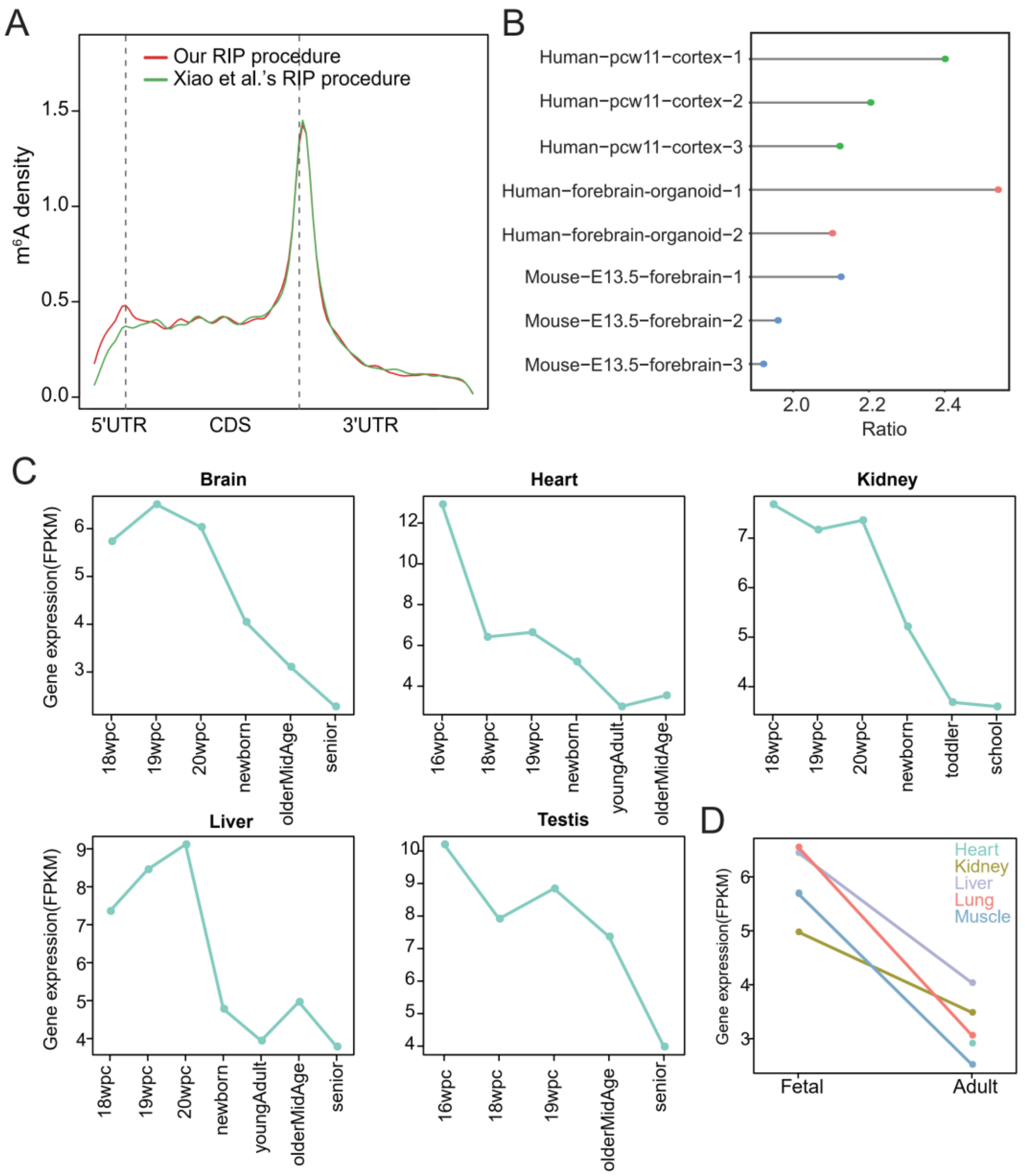

Figure S9. Analysis of m^{6}A profile and VIRMA expression level.

(**A**) The distribution of m^{6}A sites across the length of mRNA transcripts. m^{6}A-seq data were generated by two different RIP procedures and antibodies with the same HEK293T RNA sample.

(**B**) The ratio between the CDS m^{6}A site number and 3'UTR m^{6}A site number in fetal tissue m^{6}A-seq data obtained from Yoon et al. (3).

(**C**) VIRMA expression in different developmental stages in five tissues studied. Data were from Cardoso-Moreira et al (4). wpc, weeks post-conception.

(**D**) VIRMA expression in fetal and adult tissues. Fetal tissue data were from Xiao et al. (5); adult tissue data were from this study.

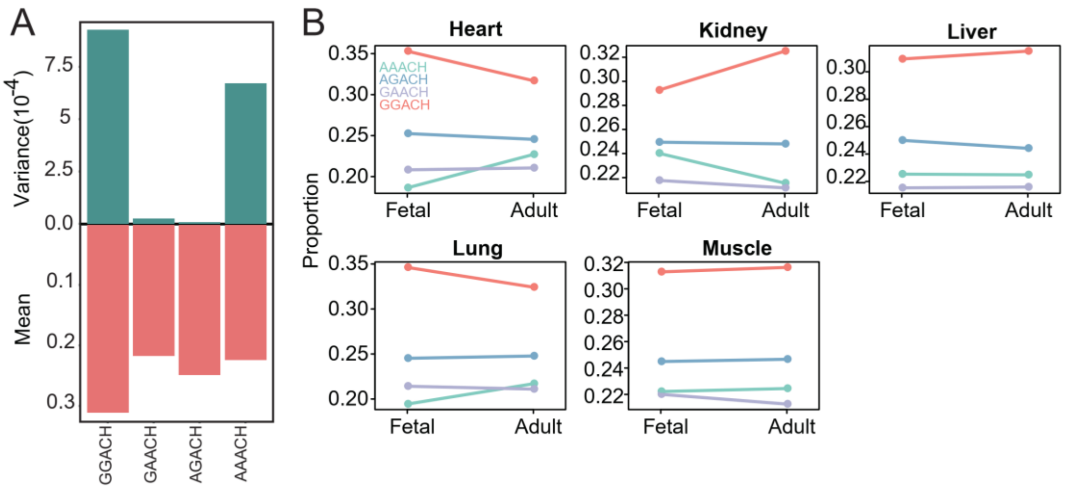

Figure S10. Dynamics of m^{6}A sub-motifs.

(**A**) Variance and mean value of the proportion of 4 sub-motifs across human fetal tissues.

(**B**) Comparison of the proportion of 4 sub-motifs between fetal and adult tissues.

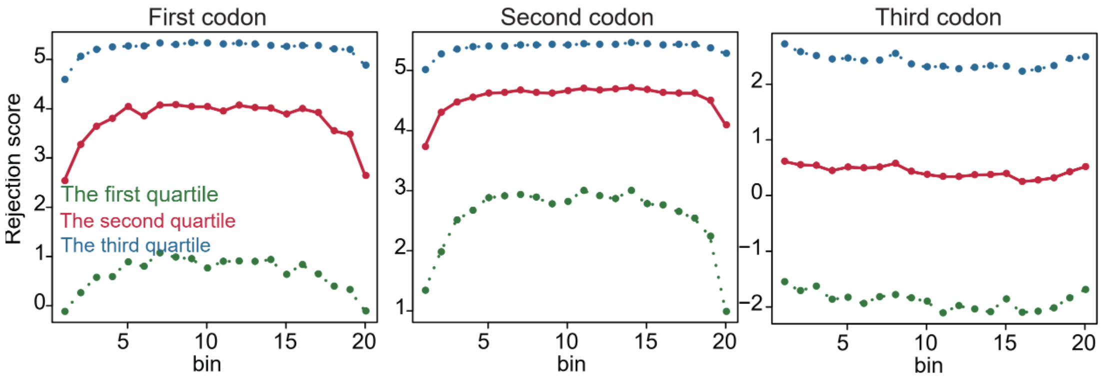

Figure S11. Rejection scores of As along the CDS regions.

As in all RRACH motif along the CDS regions were analyzed and different codon positions were plotted, separately.

REFERENCES

1. Zhang, Z., Chen, L.Q., Zhao, Y.L., Yang, C.G., Roundtree, I.A., Ren, J., Xie, W., He, C. and Luo, G.Z. (2019) Single-base mapping of m(6)A by an antibody-independent method. *Science advances*, **5**, eaax0250.
2. Linder, B., Grozhik, A.V., Olarerin-George, A.O., Meydan, C., Mason, C.E. and Jaffrey, S.R. (2015) Single-nucleotide-resolution mapping of m6A and m6Am throughout the transcriptome. *Nat Methods*, **12**, 767-772.
3. Yoon, K.-J., Ringeling, F.R., Vissers, C., Jacob, F., Pokrass, M., Jimenez-Cyrus, D., Su, Y., Kim, N.-S., Zhu, Y., Zheng, L. *et al.* (2017) Temporal Control of Mammalian Cortical Neurogenesis by m6A Methylation. *Cell*, **171**, 877-889.e817.
4. Cardoso-Moreira, M., Halbert, J., Valloton, D., Velten, B., Chen, C., Shao, Y., Liechti, A., Ascenção, K., Rummel, C., Ovchinnikova, S. *et al.* (2019) Gene expression across mammalian organ development. *Nature*, **571**, 505-509.
5. Xiao, S., Cao, S., Huang, Q., Xia, L., Deng, M., Yang, M., Jia, G., Liu, X., Shi, J., Wang, W. *et al.* (2019) The RNA N(6)-methyladenosine modification landscape of human fetal tissues. *Nature cell biology*, **21**, 651-661.
